# Effective systemic treatment of advanced periocular basal cell carcinoma with sonidegib

**DOI:** 10.1007/s00417-021-05311-z

**Published:** 2021-08-02

**Authors:** Alexander C. Rokohl, Ludwig M. Heindl

**Affiliations:** 1 Department of Ophthalmology, Faculty of Medicine and University Hospital Cologne, University of Cologne, Kerpener Strasse 62, 50937 Cologne, Germany; 2 Center for Integrated Oncology (CIO) Aachen-Bonn-Cologne-Düsseldorf, Cologne, Germany

Dear Editor,

With great interest, we read the “Letter to the Editor” of our highly appreciated colleague Nilay Yuksel. Indeed, targeted therapy for periocular and orbital tumors is becoming an exploding and hot topic for ophthalmologists and oncologists [1–5]. Sonidegib (Odomzo®) is an oral hedgehog pathway inhibitor (HPI) and that it is indicated for the treatment of adults with locally advanced basal cell carcinomas (BCCs) [2, 6, 7].

Locally advanced BCCs are defined as a subgroup of tumors that require an interdisciplinary therapeutic concept, especially in the case of an orbital involvement [5, 8]. After obtaining specific surgical and interdisciplinary expertise (tumor board), in some cases, alternative approaches to surgical excision are required since an R0 resection cannot be reliably achieved due to advanced stage of the tumor, cosmetic changes after surgery, or multiple BCC lesions [5, 9, 10]. Our patient refused any surgical or radiotherapeutic interventions due to the expected multiple surgeries as well as cosmetic disfigurements and preferred systemic treatment [2]. In addition, a reason for the patient for preferring systemic therapy was the long-term history with multiple recurrent BCCs in other locations [2]. Therefore, oral treatment with sonidegib (capsule 200 mg; Odomzo®) once a day for 6 months was administered for the treatment of the periocular BCC and for preventing potential further recurrent BCCs. Since the spread of the morphea BCC included the full width of the lower eyelid, a simple resection with ectropion surgery seemed not to be feasible [2, 9]. Rather, an extensive excision with a complex reconstruction (for example Hughes procedure) would have been necessary to achieve R0 resection with a good cosmetic result [9].

Our patient was treated successfully with sonidegib and had only slight and rarely occurring side effects not reducing significantly the quality of life [2]. However, the authors also usually recommend a surgical approach and consider the advantages of the surgery over these costly drugs, especially since the long-term efficacy and complications are not fully investigated [2, 9]. In summary with the patient’s therapy wishes and his medical history with multiple BCCs, personalized systemic therapy with the oral hedgehog pathway inhibitor (HPI) sonidegib (Odomzo®) was a good option in this case with very high patient satisfaction. Nevertheless, due to possible serious side effects, the indication for systemic treatment must always be very narrow.
